# Transcriptomic Profiling Highlights the ABA Response Role of *BnSIP1-1* in *Brassica napus*

**DOI:** 10.3390/ijms241310641

**Published:** 2023-06-26

**Authors:** Chi Zhang, Xiaoqing Yao, Yan Zhang, Shengbo Zhao, Jinghui Liu, Gang Wu, Xiaohong Yan, Junling Luo

**Affiliations:** 1 Key Laboratory of Biology and Genetic Improvement of Oil Crops of the Ministry of Agriculture and Rural Affairs, Oil Crops Research Institute, Chinese Academy of Agricultural Sciences, Wuhan 430062, China; anson-cheung@foxmail.com (C.Z.);; 2 Key Laboratory of Traceability for Agricultural Genetically Modified Organisms, Ministry of Agriculture and Rural Affairs, Wuhan 430062, China

**Keywords:** *Brassica napus*, ABA, *BnSIP1-1*, ABA transporter, signal transduction, endomembrane trafficking

## Abstract

*BnSIP1-1* is the first identified SIP1 (6b Interacting Protein1) subfamily gene of the trihelix transcription factor family from *Brassica napus (B. napus).* We previously used a reverse genetic method to reveal its abiotic stress response function in endowing plants resistance to drought and salinity, as well as ABA (Abscisic acid). However, the molecular mechanisms of *BnSIP1-1* are unclear. In this study, the global transcriptome files of *BnSIP1-1*-overexpressing transgenic and wildtype *B. napus* seedlings under ABA treatment were constructed using RNA-seq. A total of 1823 and 5512 DEGs (Differentially Expressed Genes) were identified in OE vs. WT and OE_ABA vs. WT_ABA comparison groups, which included 751 and 2567 up-regulated DEGs, and 1072 and 2945 down-regulated DEGs, separately. The impact of overexpressed *BnSIP1-1* on plants was amplified by ABA, indicating *BnSIP1-1* was an ABA-conditioned responsive gene. More interestingly, we found the reasons for *BnSIP1-1* increasing plants’ insensitivity to ABA were not by regulating ABA synthesis and catabolism, but by manipulating ABA transportation, ABA signal perception and transduction, inositol phosphate metabolism, as well as endomembrane trafficking, indirectly suggesting this gene may play roles upstream of the core ABA response pathway. Our results provided new insights into improving the knowledge about the function of *BnSIP1-1* and the ABA signaling mechanism in *B. napus.*

## 1. Introduction

With high economic value, including providing vegetable oil for human consumption and protein-rich meal for animal feed, *Brassica napus (B. napus)* is one of the most important oil crops in the world. *B. napus* is easily exposed to various abiotic stresses, such as drought and salinity, which would affect its growth and productivity. Hence, it is necessary to elucidate mechanisms of the responses of *B. napus* to diverse types of stresses and breed new varieties with higher tolerance to different growth conditions. Abscisic acid (ABA) is a plant hormone that mediates the responses to these abiotic stresses by regulating stomatal closure, gene expression, and metabolic pathways [1]. A previous study has clarified that the PYR/RCAR-PP2C-SnRK2 network is the key pathway of ABA signal transduction [2]. PYR/RCAR receptors bind ABA in a complex with ABI1 or other PP2Cs [3]. In the absence of ABA, SnRK2 protein kinase activity is inhibited by PP2C phosphatases. When ABA binds to PYR, PP2C is sequestered, resulting in activation of SnRK2 [4]. ABFs are bZIP transcription factors that bind to ABA responsive elements (ABREs) in the promoters of ABA-inducible genes and regulate their expression. SnRK2s and ABFs form a positive feedback loop that amplifies the ABA signal and enhances the stress response [5]. ABA also interacts with other hormones and signaling molecules, such as inositol phosphate [6], MAPK [7], and reactive oxygen species (ROS) [8], to fine-tune the balance between stress tolerance and growth. Several genes in *B. napus* have been reported to modulate the ABA signaling pathway. BnaA6.RGA, as a DELLA protein, positively regulates ABA signaling transduction by interacting with BnaA10.ABF2, which is a transcription factor in ABA signaling [9]. BnaABI1 is a PP2C of *B. napus* acting as a negative regulator of ABA signaling transduction. It is induced by ABA treatment and drought, then modulates stomatal closure and seed germination [10]. Although a few genes related to ABA signaling transduction have been identified, the regulation networks of these genes are still unclarified in *B. napus.*

The trihelix family is a group of plant-specific transcription factors that are characterized by a conserved trihelix DNA-binding domain. They can bind to GT elements in promoters of various genes and regulate their expression [11]. The trihelix family can be classified to five subfamilies, including GT1, GT2, SH4, GT*γ*, and SIP1 subfamilies [12]. To date, the trihelix family has been identified or expression profiled in more than 10 species [13]. For example, there are 30 and 52 trihelix genes in *Arabidopsis thaliana* (*A. thaliana*) and *Brassica rapa* (*B. rapa*) [14]. Trihelix family members are involved in many aspects of plant growth and development, such as flower formation, trichome and stomata development, seed maturation, and abscission [15,16,17]. Additionally, some trihelix family members have been reported to be involved in response to biotic and abiotic stresses. *PtrGT10 and TaGT2L1D* have been revealed to be involved in the osmotic response in poplar and wheat, respectively [18]. *TaGT2L1D*, which is a trihelix gene from wheat, negatively regulates drought resistance [19]. Several trihelix genes have been evidenced to be regulated by ABA or to participate in ABA signaling pathways. In rice, *OsGTγ-1* has been identified, which can be strongly induced by exogenous ABA [20]. *GmGT-2a* and *GmGT-2b*, which were cloned from soybean, have been proved to endow plant tolerance to abiotic stress through affecting ABA sensitivity [21]. *PgGT1* is positively regulated by ABA in *Platycodon grandifloras* [22]. Although several trihelix genes’ expression patterns in reacting to abiotic stresses or external hormones have been revealed, the functions and regulatory mechanisms of these genes under stress conditions are still largely unknown and require further investigation.

*B. napus* is an important source of edible vegetable oil and feed protein, but China has had a shortage of rapeseed and depended on imports for a long time. The study of the stress mechanism of rapeseed response to ABA could provide a new solution for improving the adaptability of rapeseed in different habitats of China. Trihelix genes’ revolutionary history among Brassica species has been explored recently. Ninety-four trihelix genes have been characterized and thirty genes belonging to the SIP1 subfamily have been identified in *B. napus*. Most trihelix genes responding to abiotic stress in *B. napus* belong to the SIP1 clade [23]. Based on our previous study, *BnSIP1-1* is a member of the SIP1 subfamily of trihelix genes expressed in various tissues of *B. napus* and can be induced by ABA or different stresses such as drought, salt, and osmotic pressure [24]. A previous study proved overexpressed *BnSIP1-1* can reduce the sensitivity of *B. napus* to ABA and positively regulate seed germination and seedling growth under stress conditions. Although *BnSIP1-1* has been reported to be involved in multiple abiotic stresses, the mechanism of *BnSIP1-1* in response to ABA stress is still unknown. Here, we performed a transcriptome analysis of *B. napus* seedlings under ABA treatment, aiming at profiling differentially expressed genes and pathways for elucidating the ABA response mechanism of *BnSIP1-1* in *B. napus.*

## 2. Results

### 2.1. Overview of Transcriptome Sequencing

To have a better understanding how *BnSIP1-1* reverses the trend of growth retardation caused by ABA in *B. napus,* tender seedlings were selected as the processing objects to obtain more sensitive data. Although there was no visible phenotype observed (Figure S1), we believed that many molecular level changes would be completely overturned based on our previous studies in *BnSIP1-1*-overexpressing *B. napus* [13,24]. Four-day-old seedlings of wildtype and transgenic plants treated with exogenous ABA were used for global transcriptomic analyses. There were 12 samples in total, which were divided into four groups: wildtype *B. napus* without exogenous ABA treatment (WT) and *BnSIP1-1*-overexpressing *B. napus* without exogenous ABA treatment (OE); WT treated by exogenous ABA (WT_ABA) and OE treated by exogenous ABA (OE_ABA). Each group included three biological replicates. Based on different background materials, these 4 groups were also divided into WT lines (WT and WT_ABA) and OE lines (OE and OE_ABA). Through the high-throughput RNA-seq method, we obtained 12 RNA-seq libraries (Table S1a), and raw reads of each group ranged from 52 to 57 million (Table 1). After removing the reads with adapters and low-quality reads, clean reads of each group numbered from 49 to 53 million (Table 1). We aligned a total of 92.14 GBase clean reads with reference sequences, with an alignment efficiency ranging from 74.55% to 76.21% and an average alignment efficiency of 75.31% (Table S1a,b). Among the four groups, the proportion of sequences with unique alignment positions on the reference genome was between 69.67% and 70.82% (Table 1).

### 2.2. Differentially Expressed Gene (DEG) Analysis

**Figure 1:**
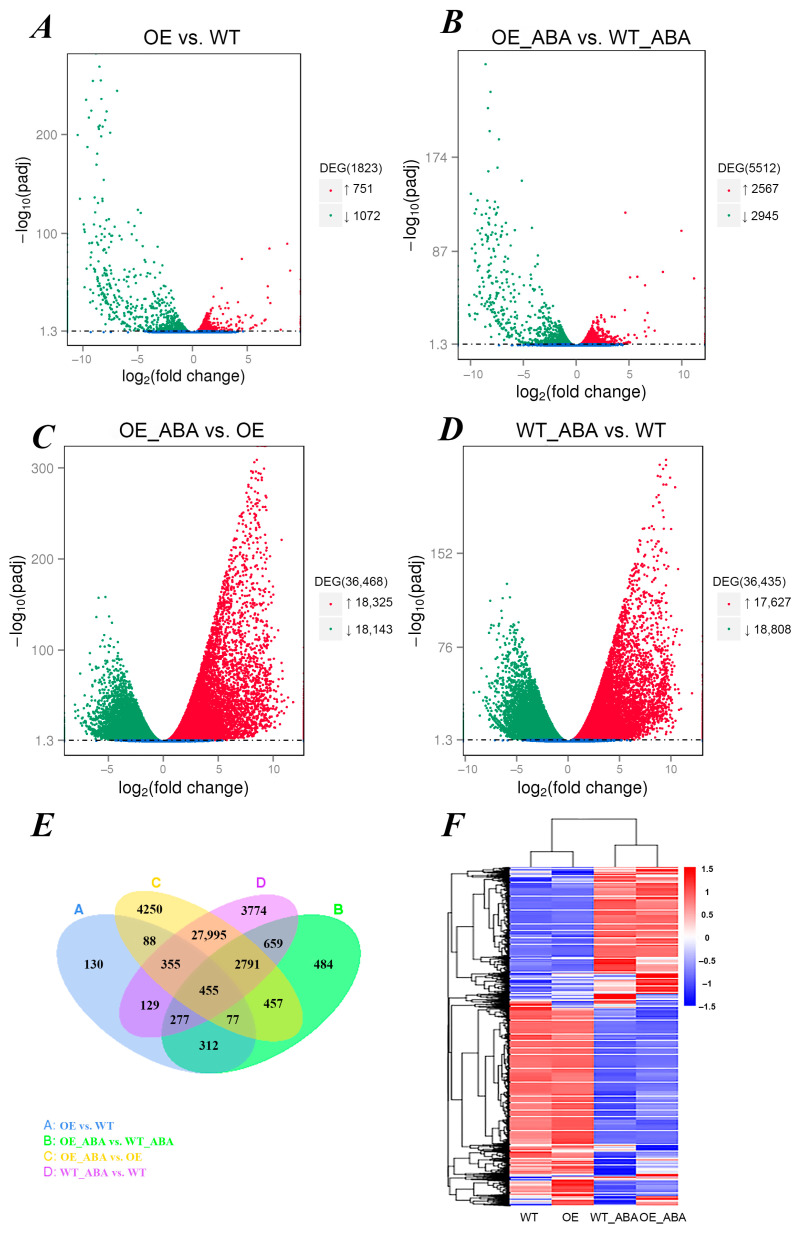
Overview of differentially expressed genes (DEGs) in transcriptome. (**A**–**D**) Genes with significant differential expression are represented by red dots (up-regulated) and green dots (down-regulated), and genes without significant differential expression are represented by blue dots; the abscissa represents the fold change of gene expression in different samples; the ordinate represents the statistical significance of the difference in expression. (**E**) Venn diagram showing the number of DEGs which were unique or shared in 4 comparison groups. (**F**) The overall FPKM hierarchical clustering map, the log_10_ (FPKM + 1) value was normalized and clustered, red indicates highly expressed genes and blue indicated poorly expressed genes. The color change from red to blue indicates a descending log_10_ (FPKM + 1) value.

A total of 1823 and 5512 DEGs were identified in OE vs. WT and OE_ABA vs. WT_ABA comparison groups, which included 751 and 2567 up-regulated DEGs, and 1072 and 2945 down-regulated DEGs, separately (Figure 1A,B,E). These DEGs can be defined as “*BnSIP1-1* affected” because they received the same treatment but had significantly different expression levels only because *BnSIP1-1* overexpressed or not. Similarly, a total of 36,468 and 36,435 DEGs were identified in OE_ABA vs. OE and WT_ABA vs. WT groups, including 18,325 and 17,627 up-regulated DEGs and 18,143 and 18,808 down-regulated DEGs, separately. These DEGs can be defined as “ABA affected” because the appearance of such DEGs was only caused by the application of exogenous ABA, not other factors (Figure 1C,D). The number of DEGs in different comparisons could be used to evaluate the variable-associated responsive degree of *B. napus.* It was found that ABA treatment amplified the numbers of “*BnSIP1-1*-affected” DEGs by several times (up-regulated: 3.41; down-regulated: 2.74) (Figure 1A,B). Meanwhile, the DEGs affected by ABA showed larger variation with more significant differences in the OE_ABA line than in the WT_ABA line, indicating *BnSIP1-1* was involved in ABA response (Figure 1C,D). By performing a hierarchical cluster analysis, we found that in the WT and OE group the gene expression pattern was similar, while the gene expression patterns of OE_ABA and WT_ABA were apparently different in the whole transcriptome (Figure 1F). These results not only indicated that *BnSIP1-1* was related to ABA but also suggested the impact of overexpressed *BnSIP1-1* on plants would be amplified by ABA.

### 2.3. GO Enrichment of DEGs

**Figure 2:**
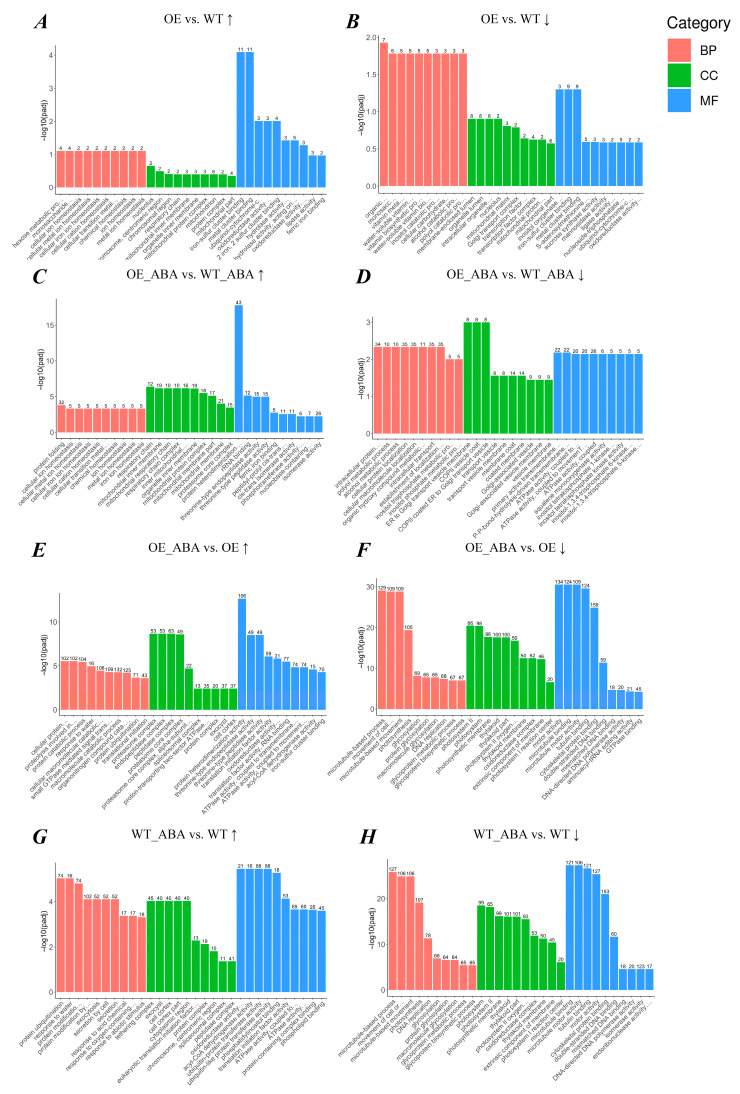
GO enrichment histogram of DEGs. The top 10 significantly enriched GO terms in each GO term category obtained from pairwise transcriptome comparison. If there are fewer than 10 terms per category, all terms are displayed. The ordinate was –log_10_ (padj), which refers to adjusted *p*-value after multiple hypothesis testing correction. The value range of padj is [0, 1], and the closer to zero, the more significant the enrichment. The abscissa is the description of the GO term. The number of differential genes in the specific GO term is shown above each bar. Different colors are used to distinguish biological processes, cellular components, and molecular functions. (**A**–**H**) The histogram of DEGs from 4 pairwise comparison groups. “↑” represents the up-regulated DEGs, “↓” represents the down-regulated DEGs.

Gene ontology enrichment analysis clarified the manifestation of sample differences in gene function in the experiment. By performing a GO enrichment analysis on DEGs, we found there were 52,583 genes which can be annotated with 249 GO terms. Among them, the ones that belonged to the catalytic complex had the largest portion, numbering 771. For “*BnSIP1-1*-affected” genes, drug metabolic process was the most significant enriched GO term (54 DEGs). For “ABA-affected” genes, the most significantly enriched one was catalytic complex (276 DEGs). In addition, we found there was no apparently enriched GO term in the OE vs. WT group except terms of iron–sulfur cluster binding and metal cluster binding, suggesting overexpression of *BnSIP1-1* had little influence on plants under normal growth conditions (Figure 2A,B). After ABA treatment, the up-regulated “*BnSIP1-1*-affected” DEGs were significantly assigned to the GO terms of protein folding, proteasome core complex, and protein heterodimerization activity with a significant difference between OE_ABA and WT_ABA plants (Figure 2C). Meanwhile, the down-regulated “*BnSIP1-1*-affected” genes related to endomembrane trafficking, such as Golgi-associated vesicle, COPII vesicle, and COPII-coated ER to Golgi transport vesicle, were more notable in the comparison of OE_ABA vs. WT_ABA than in the comparison of OE vs. WT (Figure 2D–F). ABA treatment affected genes involved in inositol phosphate metabolism and ATPase activity, which are known to be influenced by ABA. These genes showed more significant differences in the OE line than in the WT line (Figure 2E–H). Based on these results, we could infer that *BnSIP1-1* may regulate endomembrane trafficking and be involved in the biological process that ensures proper protein folding in the cytoplasm.

### 2.4. KEGG Enrichment of DEGs

**Figure 3:**
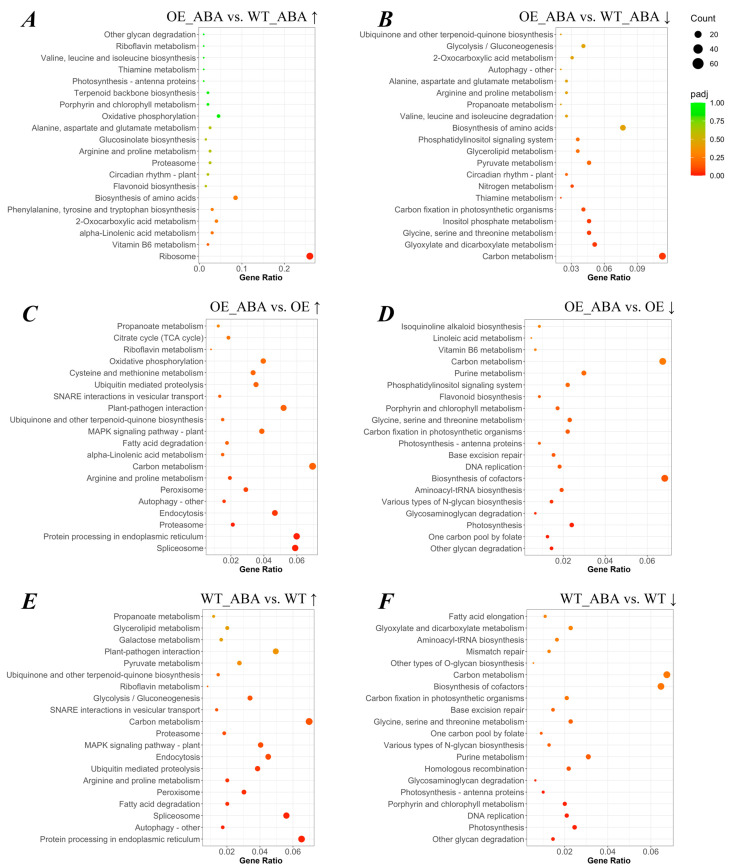
KEGG enrichment scatter plot of DEGs. The vertical axis represents the name of the pathway, the horizontal axis represents the gene ratio, the size of the points represents the number of DEGs in this pathway, and the color of the points corresponds to different adjusted *p*-value ranges. Gene ratio refers to the ratio of the number of differential genes enriched in the pathway (number of sample genes) to the number of annotated genes (number of background genes). The larger the enrichment factor, the greater the degree of enrichment. Adjusted *p*-value (padj) is the *p*-value after multiple hypothesis testing correction. The value range of padj is [0, 1]. The closer to zero, the more significant the enrichment. We selected the 20 most enriched pathways to display in this figure, and if there are fewer than 20 enriched pathway entries, all are displayed. (**A**–**F**) The scatter plot of up-regulated and down-regulated DEGs in different comparison groups mentioned above. “↑” represents the up-regulated DEGs, “↓” represents the down-regulated DEGs.

Significant enrichment of KEGG pathways can determine the most important biochemical metabolic pathways and signaling pathways that DEGs participate in. Among all transcriptome data, 39,510 genes could be annotated with 117 KEGG terms. The term including the largest number of DEGs (278) was hormone signal transduction. We found the number (*n* = 52) of DEGs annotated as ribosome was the greatest in the “*BnSIP1-1*-affected” group (Figure S2A,B), while carbon metabolism had the most DEGs annotated among the “ABA-affected” group, with 150 genes annotated (Figure S2C,D). Exogenous ABA up-regulated the genes related to ubiquitin-mediated proteolysis in both WT plants and OE plants; meanwhile, this KEGG term was more enriched in the WT line than the OE line, with *p*-values of 0.004 and 0.065, respectively (Figure 3C,E). The down-regulated “*BnSIP1-1*-affected” DEGs which were assigned to circadian rhythm, inositol phosphate metabolism, phosphatidylinositol signaling system, and autophagy had a great degree of enrichment after ABA treatment because of the superposition effect of ABA and *BnSIP1-1* (Figure 3B). DEGs involved in the phosphatidylinositol signaling system and isoquinoline alkaloid biosynthesis had a lower expression level after ABA treatment in OE plants than in WT plants (Figure 3D). There were no significantly enriched terms identified in the comparison of OE vs. WT by KEGG analysis, so the data of this comparison are not presented in Figure 3. Taken together, KEGG analysis indicated *BnSIP1-1* may affect ABA-related genes by regulating the activity of molecules which are involved in different pathways, including ubiquitin-mediated proteolysis, inositol phosphate metabolism, the phosphatidylinositol signaling system, and autophagy.

### 2.5. Analysis of the Transcription Factor in DEGs

In all the unique DEGs obtained from 4 pairwise comparisons, 1456 genes belonging to 72 different TF families were found via iTAK software analysis (Table S2). The largest portion of these TFs belonged to the AP2-EREBP family, followed by MYB and NAC families. The numbers of TFs belonging to specific TF families ranged from 1 to 134 (Table S2). Exogenous ABA resulted in different expression patterns of TF genes in both wildtype and transgenic materials (Table 2). The comparison of OE_ABA vs. WT_ABA showed a significant increase in the number of DEGs in all TF families compared to the comparison of OE vs. WT (Table 2). In this comparison, the number of DEGs belonging to AP2-EREBP was greater than in other TF families, while under a normal growth situation, overexpression of *BnSIP1-1* mainly affected the TF genes classified into the MYB family (Table 2). These results suggested that, to respond to exogenous ABA, *BnSIP1-1* may affect downstream genes indirectly by regulating other TF families, such as MYB and AP2-EREBP, and ABA could increase the effect caused by the overexpression of *BnSIP1-1.*

### 2.6. Validation of RNA-Seq by RT-qPCR

**Figure 4:**
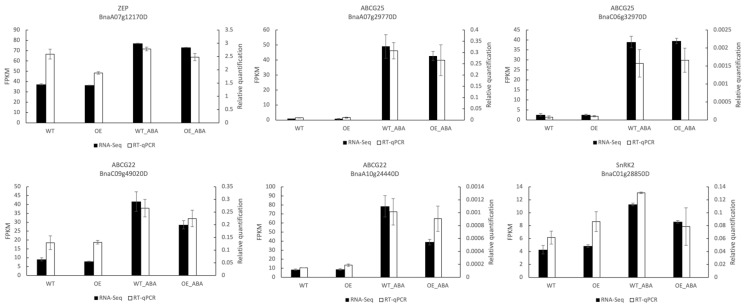
Expression pattern of six DEGs by RNA-seq and RT-qPCR. Black bars represent the expression level changes based on FPKM values of RNA-seq (left *y*-axis). White bars represent the expression levels detected by RT-qPCR (right *y*-axis). Error bars indicate the standard errors of the means (*n* = 3).

To verify the accuracy of RNA-seq, six genes were selected for RT-qPCR assay. These selected genes were related to ABA metabolism and transport, including one ABA synthesis *ZEP* (BnaA07g12170D), two ABA exporter *ABCG25* (BnaA07g29770D and BnaC06g32970D), two ABA importer *ABCG22* (BnaA10g24440D and BnaC09g49020D), and one serine/threonine protein kinase gene *SnRK2* (BnaC01g28850D). The RNA sample was obtained in the same way as those used in RNA-seq. The results indicated that change pattern of the selected genes was similar between RNA-seq and RT-qPCR, confirming the validity and reliability of our RNA-seq data and our findings (Figure 4).

## 3. Discussion

### 3.1. ABA Metabolism and Signaling System in OE Plants

In general, ABA content in plants is determined by synthesis and catalysis, however, ABA transportation from the external environment should also be considered as an important effector of inner ABA content when externally treated with ABA [25]. Based on our previous study (Figure 8A of [24]), the ABA content of leaves was slightly higher in OE seedlings than in WT seedlings without any treatment, afterwards, when treated with exogenous ABA, the content of ABA in both OE and WT leaves began to increase, but the increase rate of ABA content in OE was higher than that in WT, resulting in the content of ABA in leaves gradually becoming equal to WT (0-6 h treatment) and even surpassing WT with the extension of ABA treatment time (24 h treatment) [24]. The fine dynamic changes in ABA content in transgenic plants undergoing ABA treatment fully indicated that *BnSIP1-1* was involved in ABA response. 

**Figure 5:**
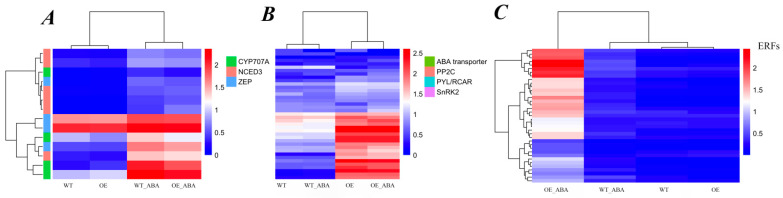
Hierarchical cluster analysis of ABA-related genes in transcriptome. The FPKM value was normalized by log_10_ (FPKM + 1) and clustered. Red indicates highly expressed genes and blue indicates poorly expressed genes. The color change from red to blue indicates a descending gene expression level. (**A**) Expression pattern of genes related to ABA metabolism and biosynthesis. (**B**) Expression pattern of genes related to ABA transport and signaling pathway. (**C**) Expression pattern of ERFs (Ethylene Response Factors) found in OE and WT with or without ABA treatment.

As we know, early biosynthesis steps of ABA are catalyzed by zeaxanthin epoxidase (ZEP/ABA1) and 9-cis-epoxycarotenoid dioxygenase (NCED) enzymes, and the oxidative catabolism of ABA is mainly performed by *CYP707A*, which encodes an ABA 8′-hydroxylase of the P450 gene family [26]. To reveal the ABA response mechanism of *BnSIP1-1*, we first focused on the analysis of DEGs involved in ABA biosynthesis and catalysis. There were ten ABA synthesis-related and four catabolism-related DEGs identified in these transcriptome data, including ZEP/ABA1, NCED3, and CYP707A1/2/4. Most of the expression levels of these genes did not show any changes when untreated, except *CYP707A2* (BnaA04g16900D), which deceased about 40% in OE plants compared with WT plants (Figure 5A, Table S3). Consequently, this gene’s down-regulation may result in the decrease in oxidative inactivation of ABA, leading to a slightly higher ABA content in OE plants than in WT plants. After ABA treatment, although all of the DEGs related to ABA synthesis and catabolism were up-regulated in both plants, the increase in two *CYP707A1* (BnaA03g43960D and BnaC07g35800D) genes and one *CYP707A2* (BnaA04g16900D) gene in OE_ABA samples was much less than that in WT_ABA samples, with the former only about half of the latter (Table S3a), which can provide a good explanation for why ABA content increased faster in OE than in WT plants after ABA treatment, as revealed by our previous study [24]. However, there was an obvious contradiction regarding the increase in ABA content and insensitivity of ABA response in OE plants treated by external ABA. Therefore, we focused on DEG-related ABA transportation. Interestingly, three ABA exporter *ABCG25* (BnaA07g29760D, BnaA07g29770D, and BnaC06g32970D) and two ABA importer *ABCG22* (BnaA10g24440D and BnaC09g49020D) [25] genes were significantly up-regulated in both OE_ABA vs. OE and WT_ABA vs. WT comparisons, however, only the two importers of *ABCG22* were down-regulated in OE_ABA plants compared with WT_ABA plants (Figure 5B, Table S3a), suggesting *BnSIP1-1* may help reduce the cell’s sensitivity to exogenous ABA by preventing excessive ABA being delivered into guard cells by *ABCG22*. This finding further confirmed that fine regulation of ABA transport helps plants cope with abiotic stress. 

The PYR/RCAR-PP2C-SnRK2 pathway is the core of ABA signal transduction. PYR/RCAR receptors bind ABA, negatively regulating the activity of ABI or other PP2Cs, which could inhibit the protein kinase activity of SnRK2s to reduce the ABA signal and decrease stress response [2,27]. In this study, seventeen *PP2C* genes, fourteen *PYR/RCAR* genes, and three *SnRK2* genes were identified as DEGs (Table S3b, Figure 5B). Most of these genes were up-regulated in both OE and WT plants after treatment with ABA. The different change patterns of ABA signal-related DEGs between OE_ABA and WT_ABA were the most important hint for revealing the relationship of *BnSIP1-1* and the ABA response network. Four *PYR/RCAR* genes, including one *PYL5* (BnaAnng40650D), one *PYL7* (BnaC03g31730D), and two *RCAR1s* (BnaC06g17940D and BnaA10g00540D), were up-regulated and only one *PYL5* (BnaC03g02130D) was down-regulated in OE_ABA plants compared with WT_ABA plants, which indicated that *BnSIP1-1* rapidly transmitted ABA signals by selectively accelerating (compared to WT) the up-regulation of certain ABA core receptors, thereby accelerating the response of downstream genes. There were one *ABI1* (BnaC01g18020D), two *ABI2* (BnaA10g11080D and BnaC09g53650D), and one *SnRK2* (BnaC01g28850D) genes that were down-regulated in the comparison of OE_ABA vs. WT_ABA. Although *ABI1/2* act as negative regulators in ABA signal transduction, their function cannot be simply defined. The mutant of *ABI1* and the mutant of *ABI2* share a common ABA-insensitive phenotype because of the lower activity of phosphatase and affinity of Mg^2+^ [28], indicating dynamic balance or fine conditioning of *ABI1/2* may be essential for maintaining their negative regulatory function in the cell. Overall, these results indicated that *BnSIP1-1* mainly inhibited ABA signaling by accelerating the expression of *PYR/RCAR*-related genes and reducing the content of *SnRK2s*, as well as fine regulation of *ABI1/2* to blunt the sensitivity of plants to ABA. 

### 3.2. Transcription Factor Analysis

Many transcription factors have been reported to play essential roles in plant ABA response. Among 1456 genes from 74 TF families showing changed expression patterns, 257 genes in 42 families were found presenting significant differences in comparisons between OE_ABA vs. WT_ABA, although only 70 genes in 30 families were identified as changed in comparison between OE vs. WT. These data indicated *BnSIP1-1* responded to ABA by regulating a wide range of genes in different TF gene families, especially gene families AP2-ERFBP, MYB, HB, NAC, C2H2, Tify, WRKY, and ZIP. The results of this study supported those of previous research (Table 2, Figure 5C and Table S2). 

Our data showed that, in both OE and WT lines, most TF gene family members were up-regulated after ABA treatment, except zf-HD and bHLH families, indicating *B. napus* leaves respond to ABA mainly by positively regulating the expression of transcription factors (Table S2). Moreover, among all the DEGs in comparisons of OE_ABA vs. WT_ABA, there were 149 up-regulated and 108 down-regulated genes, which also meant that *BnSIP1-1* responded to ABA stress by positively regulating transcription factors to a greater extent. The most typical example was the AP2-EREB TF family, with 3 times more up-regulated (*n* = 29) than down-regulated (*n* = 8) genes in OE_ABA vs. WT_ABA comparison groups (Table S2). The AP2-EREB TF family is a big family including AP2 (APETALA2), DREB, ERF, RAV, and Soloist subfamilies [29]. Members of the ERF subfamily were the most abundant (*n* = 23) among all the AP2-EREB TF family DEGs (*n* = 37). Based on the hierarchical cluster analysis of the ERF subfamily, we found most ERF DEGs up-regulated in the comparison of OE_ABA vs. WT_ABA (Figure 5C). Genes belonging to this subfamily are known as ethylene response factor (*ERF*) genes. Our results suggest that *BnSIP1-1* overexpression changes the expression of *ERF* genes that are involved in the crosstalk of ABA and ethylene (Table 2 and Table S2, Figure 5C). Based on a previous study, the induced expression of specific *ERFs* can lead to an ABA-insensitive phenotype in *A. thaliana* [30], which was consistent with our present study. The WRKY family is a widely reported TF family related to ABA response. In our results, there were 70 WRKY DEGs identified in OE_ABA vs. OE, and 11 WRKY DEGs had differential expression levels between OE_ABA and WT_ABA. Among them, eight DEGs were up-regulated, such as *WRKY28* and *WRKY40*, and three DEGs were down-regulated, such as *WRKY2* and *WRKY11* (Table S2). Notably, there were three *WRKY40* (BnaA07g20110D, BnaAnng23990D, and BnaC02g26030D) genes showing consistently increased expression in OE_ABA compared with WT_ABA. In *A. thaliana, WRKY40* directly leads to the repression of *ABI5* [31]. The mutant of *abi5* has been characterized as having ABA insensitivity characteristics [32]. In the current study, *WRKY40* was defined as an “ABA-affected” as well as “*BnSIP1-1*-affected” DEG. *WRKY40* expression increased in both OE and WT plants after ABA exposure, but the increase was stronger in OE plants than in WT plants. *ABI5* (BnaA05g08020D) had the opposite expression profile to *WRKY40* in these transcriptome data, which showed down-regulation in OE_ABA vs. WT_ABA (Table S2). This result agreed with a previous study that *WRKY40* is the negative regulator of *ABI5*, suggesting a tight relationship between *BnSIP1-1* and *WRKY40*. Taken together, we assumed the *BnSIP1-1* regulation network may have crosstalk with extensive transcriptional regulatory pathways, including the *ERF* regulation network, WRKY family, and others, thus regulating downstream ABA-responsive genes via the ABA signal pathway and other hormone signal pathways (e.g., ethylene).

### 3.3. Inositol Phosphate Synthesis and Catabolism in OE Plants

Inositol metabolism has been proved to play an important role in ABA signaling transduction [6,33,34,35]. Among “*BnSIP1-1*-affected” DEGs, there were eight genes that were annotated with the KEGG term inositol phosphate metabolism. Among these eight DEGs, three DEGs were discovered in OE vs. WT groups, indicating that, regardless of ABA exposure, *BnSIP1-1* was correlated with IP metabolism. Meanwhile, seven of eight DEGs had been found in OE_ABA vs. WT_ABA groups, proving ABA exposure also amplified the effect of *BnSIP1-1* overexpression in IP metabolism. Additionally, six genes’ expression patterns were affected by exogenous ABA in OE plants, while eight genes’ expression patterns were affected by ABA in WT plants. This result suggested that, in both plants, the genes related to IP metabolism had crosstalk with the ABA pathway, which agreed with the previous studies mentioned above [6,33,34,35,36]. *FRY1* (also as known as *SAL1*) is a bifunctional enzyme, encoding inositol polyphosphate 1-phosphatase, which is involved in inositol catabolism [33]. The mutant of *FRY1* not only increases the accumulation of IP_3,_ but also makes the process of seed germination more sensitive to exogenous ABA, suggesting *SAL1* is a negative regulator in the ABA signaling pathway [33]. In this study, the expression level of *SAL1* (BnaA09g06680D) was down-regulated by the ABA treatment in two background materials. However, in the OE_ABA group, *SAL1* showed less variation and had a significantly higher expression level than in the WT_ABA group, which may reduce the sensitivity of *B. napus* to ABA. Overall, *BnSIP1-1* may play a role as a vital regulator in IP metabolism, leading to a higher turnover rate of inositol phosphates to reduce ABA sensitivity in *B. napus.*

### 3.4. DEGs Related to Endomembrane Trafficking

Endomembrane trafficking functions in sorting and transporting of materials between different organelles within cells. The tight link between endomembrane trafficking and abiotic stress to ensure the correct delivery of stress-related cargo molecules has been supported by previous studies [37]. However, the underlying mechanisms remain unknown. DEGs involved in endomembrane trafficking were enriched based on GO analysis in this study. These genes’ expression patterns did not show any difference between OE and WT plants, however, after treatment with ABA, one *SAR1* gene was up-regulated and eight *Sec23/Sec24* genes were down-regulated in the comparison between OE_ABA vs. WT_ABA, indicating *BnSIP1-1* may inhibit transport of molecules within cells through vesicle sorting. The soluble *N*-ethylmaleimide-sensitive factor attachment protein receptor (SNARE) family is a protein family that has been characterized as the key component driving membrane fusion [38,39,40]. *VAMP721/722* is a subunit of the ternary SNARE complex [41]. In *A. thaliana*, a previous study elucidated ABA-induced plant growth retardation is partly caused by the reduced level of *VAMP721/722*, which would limit the efficiency of transportation in the plasma membrane and cell wall [42]. Here, we found the transcription of *VAMP722* (*SAR1*, BnaA09g50530D), homologous to Arabidopsis *VAMP721*, in OE_ABA was significantly higher than in WT_ABA (Table S5), indicating *BnSIP1-1* may impede the ABA-induced depletion of *VAMP722* to improve efficient sorting within cells. *Sec23/Sec24* are two proteins that form a complex that acts as the inner coat of COPII, which is a complex vesicle that mediates the ER–Golgi trafficking process. Mutants of *AtSec24* can be fatal for plants [43]. In our transcriptome, eight genes encoding *Sec23/Sec24* had lower expression in OE than in WT after ABA treatment (Table S5). Genetic, biochemical, and structural studies have confirmed a series of interactions between cargo proteins and *Sec24*, revealing the basis of cargo specificity in COPII vesicle formation. Yeast Sec24 has at least four distinct cargo protein loading sites that recognize different sorting signals, thus diversifying the range of cargo that can be exported by the same vesicle [44,45]. The diversity of cargo output can be further achieved by combining Sec24 subunits expressing different forms. Examples include yeast expressions (Iss1/Sfb2 and Lst1/Sfb3) and mammalian expressions (Sec24A/B and Sec24C/D), each with different cargo specificities [44,45]. It is still a great mystery why so many *sec23/24* coding genes were down-regulated relative to wildtype in transgenic materials when treated with ABA, because the specific sorting rules of sec23/24 for cargo molecules in plants are not clear [46,47]. The mechanism regulating the turnover of PYR/PYL/RCAR-type receptor-ABA-PP2C complexes and endosomal trafficking has recently drawn attention, especially regarding the regulatory role of ubiquitination-associated membrane trafficking [48]. Overall, these results implied that *BnSIP1-1* mainly negatively regulates ER–Golgi trafficking, repressing the sorting and transferring of ER stress-related genes to impede ABA signaling transmission. 

### 3.5. DEGs Related to MAPK Signaling

The MAPK signaling cascade pathway is widely involved in multiple development stages of plants. Recently, several MAPK family members have been reported to be directly or indirectly activated by the ABA pathway [7]. In this study, our data indicated that one hundred MAPK members are modulated under ABA treatment (Table S6). There were five up-regulated and ten down-regulated MAPK members identified in the comparison of OE_ABA vs. WT_ABA groups, hinting that these genes respond to ABA stress by regulating the MAPK signaling pathway through negative feedback. The genes encoding *MAP3K3/14/17/18* (BnaA03g38420D, BnaA10g27890D, BnaC04g40820D, BnaCnng21650D, BnaAnng12350D), *MAP2K5* (BnaA01g25260D), and *MAPK8* (BnaA09g44690D) were apparently activated by ABA, while in the OE line, the change in expression profile was more gradual than in WT line. Previous evidence suggested *ABI1,* which is a member of *PP2C* in the ABA signaling pathway, can regulate the activity of *MAP3K18* in *A. thaliana* [49]. In our data, we found that a PP2C gene, *ABI1*, and a *SnRK2* gene, *SnRK2B*, shared an expression pattern with the gene encoding *MAP3K18* (Table S6). These results agreed with a previous genetic study’s conclusion that the *MAP3K18* signaling pathway is regulated by the PYR/RACR-PP2C-SnRK2 pathway [50]. The result also implied that the overexpression of *BnSIP1-1* may negatively regulate these six genes with a similar pattern and reduce the accumulation of MAPK members, thereby inhibiting downstream ABA-induced genes.

MAPK cascade can be activated not only by ABA but also by ROS [51]. Hydrogen peroxide (H_2_O_2_) is a form of ROS. The interaction of *RbohD* and *RbohF* directly generates superoxide anions in apoplasts and finally convert them to H_2_O_2_. The *RbohD* (BnaA06g30520D, BnaAnng07990D, BnaC02g38300D, and BnaC07g26270D) and *RbohF* (BnaA09g11870D, BnaC09g12490D) genes we identified in the transcriptome were both up-regulated in response to ABA in the OE line and WT line (Table S7), which meant these genes were defined as “ABA-affected” DEGs. The increased activity of *RbohD* and *RbohF* inevitably leads to ROS accumulation and activates the MAPK cascade. Noticeably, *RbohF in* OE_ABA was expressed more weakly than in the WT_ABA group (Table S7), which signified that it had a similar expression pattern to *MAP3K18* in the current transcriptome, indicating *BnSIP1-1* may be responsible for the reduction of the accumulated ROS and indirectly moderate gene expression following the MAPK pathway.

## 4. Material and Methods

### 4.1. Materials and Growth Conditions

*Brassica napus* cv. ZhongShuang 6 (ZS6) was defined as wildtype and the transgenic *B. napus* was generated according to a previously reported method [24]. Positive T_3_ generation plants were grown to produce cDNA libraries. The transgenic lines OE-7 and OE-27 overexpressing *BnSIP1-1* were used for this study, in order to maintain consistency with previous research [13,24]. Lines OE-7 and OE-27 were used for phenotype characterization. Line OE-7 (pool of more than 30 seedlings) was used for RT-qPCR and RNA-seq. The *B. napus* line ZS6 and the transgenic plants were sown in 1/2 Hoagland’s nutrient medium and grown in a growth chamber (22 °C with 16h light/8h dark cycle, 40-65% humidity, and light intensity of 8000 lux) for 4 days. After 4 days of cultivation, seedlings of OE and WT were treated by ABA for 6h for phenotype characterization, RT-qPCR, and transcriptome analysis.

### 4.2. RNA Sequencing

Four-day-old seedlings treated with 10 mM ABA for 6 h were selected for further transcriptome analyses. Three biological replicates were sampled for each group (WT, OE, WT_ABA, OE_ABA). A total of 12 samples of leaf tissue (more than 30 seedlings per sample) were collected with the same growth status and stored at −80 °C for further experiments. Total RNA was isolated by using TRIzol reagent (Invitrogen, Burlington, ON, Canada). Preparation of libraries and sequencing of transcriptomes were carried out using an Illumina HiSeq X Ten (Novogene, Beijing, China). 

### 4.3. Alignment of Transcriptome Data and Gene Expression Analysis

Clean reads were obtained by removing the reads with adapters, the reads for which N (N indicates that the base information cannot be determined) was greater than 10%, and the low-quality reads (with bases with a quality value Q_phred_ ≤ 20 accounting for more than 50% of the entire read) from raw reads. Clean reads were mapped to the *Brassica napus* reference genome (https://www.genoscope.cns.fr/brassicanapus/data, accessed on 3 June 2015) by TopHat2 [52]. Based on the Poisson distribution model, we standardized the processed read count data, calculated the hypothesis test probability (*p*-value) through DESeq [53], and finally performed multiple hypothesis test corrections to obtain the false discovery rate (FDR) value. DEGs were those genes with FDR-adjusted *p*-value (q-value) < 0.05. Relative expression levels were normalized in terms of fragments per kilobase of exon per million reads (FPKM). 

### 4.4. GO and KEGG Enrichment Analysis

We performed a GO enrichment analysis on DEGs selected from the above pairwise comparison via the InterProScan software [54]. In this way, the GO enrichment analysis was based on the Wallenius non-central hyper-geometric distribution. KOBAS software (KOBAS, Surrey, UK) was used to conduct KEGG pathway enrichment analysis, which can calculate the total number of DEGs involved in specific pathways [55]. All full-length transcripts were subsequently annotated using three public databases, including the NCBI Swissport, GO, and KEGG databases.

### 4.5. RT-qPCR for Validation

Real-time quantitative PCR (RT-qPCR) was applied to quantify the gene expression levels to validate the accuracy of RNA-seq data. There were 6 genes, including 1 zeaxanthin epoxidase, 2 ABA importers, 2 ABA exporters, and 1 serine/threonine-protein kinase gene. Three independent biological replicates were conducted, and each biological group was repeated three times. Plant materials were collected in the same way as the materials used in RNA-seq. RNA extraction was carried out using an RNAprep Pure Plant Kit (TIANGEN Biotech, Beijing, China). Total RNA was transcribed into cDNA by using a One-Step RT-PCR Kit (Novoprotein Scientific, Beijing, China). The qPCR was performed by using ChamQ Universal SYBR qPCR Master Mix (Vazyme Biotech, Nanjing, China) in 20 µL of reaction mixture, including: 10 µL of 2 × mix, 1 µL of each PCR forward and reverse primer for the selected gene, 4 µL of cDNA template, and 5 µL of dd H_2_O. RT-qPCR was performed on the CFX Connect real-time PCR platform (Bio-Rad, Hercules, CA, USA). The running condition of RT-qPCR was one cycle of 95 °C for 30 s, followed by 40 cycles of 95 °C for 30 s and 56 °C for 30 s. The transcription level of genes was measured by the $2^{-∆∆Ct}$ method [56]. The β-actin gene of *B. napus* was used as a reference gene. All primers were designed via Primer-BLAST [57] and are shown in Table S8.

## 5. Conclusions

In this study, we presented a comprehensive transcriptome analysis of *B. napus* leaves overexpressing *BnSIP1-1* in response to ABA, expecting to excavate the core mechanism of *BnSIP1-1* driving ABA insensitivity. A total of 1823 and 5512 DEGs were identified in OE vs. WT and OE_ABA vs. WT_ABA comparison groups, which included 751 and 2567 up-regulated DEGs, and 1072 and 2945 down-regulated DEGs, separately, indicating *BnSIP1-1* was an ABA-conditioned responsive gene. Based on clustering and GO and KEGG enrichment analysis, we conducted detailed research on enriched terms of ABA metabolism, transport, and signaling, inositol phosphate metabolism, endomembrane trafficking, MAPK signaling, ROS, and transcription factor families. Strong evidence suggested that *BnSIP1-1* played vital roles in ABA transportation rather than ABA metabolism. In addition, *BnSIP1-1* perhaps transmitted ABA signals mainly by positive regulation of ABA upstream signals, leading to their rapid conduction, such as the PYR/RCAR-PP2C-SnRK2 pathway, but caused negative regulation of downstream signal amplification pathways, such MAPK signaling. For downstream effector genes rather than regulatory genes, their regulation by *BnSIP1-1* reflected diversity, such as extensive up-regulation of a large number of ERF transcription factor genes, as well as down-regulation of other genes of different pathways. These results provided a broader and better understanding of the molecular bases of a single transcription factor responding to ABA by causing changes in the comprehensive transcriptional spectrum of ABA signaling upstream and downstream, as well as effector genes of the response chain terminal. Our study will help to develop a better understanding of abiotic stress-related mechanisms and the broad functions of transcriptional regulatory networks in *B. napus* in general.

## Figures and Tables

**Table 1:** Summary of RNA-seq data.

Sample	Raw Reads	Clean Reads	Uniquely Mapped Reads
WT	55,155,064	51,801,457 (93.92%)	36,088,213 (69.67%)
OE	57,301,865	53,917,446 (94.09%)	37,586,943 (69.71%)
WT_ABA	54,124,437	50,870,907 (93.99%)	36,028,550 (70.82%)
OE_ABA	52,437,185	49,498,073 (94.39%)	34,923,275 (70.55%)

**Table 2:** Distribution of differentially expressed TFs.

TF Family	OE vs. WT	OE_ABA vs. WT_ABA	OE_ABA vs. OE	WT_ABA vs. WT
ABI3VP1	0	0	14	11
Alfin-like	0	0	6	5
ARID	0	0	1	1
ARR-B	0	0	2	2
BBR/BPC	1	0	7	7
BSD	1	0	4	3
C2C2-GATA	1	0	20	18
CAMTA	0	0	9	9
CPP	0	0	3	3
DBP	0	0	2	2
DDT	1	0	3	3
EIL	0	0	9	9
FAR1	0	0	1	1
FHA	0	0	9	9
GNAT	0	0	20	20
IWS1	1	0	8	8
LIM	0	0	1	1
LUG	0	0	2	1
MBF1	0	0	6	5
MED7	0	0	3	3
mTERF	0	0	2	3
OFP	0	0	3	3
PLATZ	0	0	17	16
S1Fa-like	0	0	2	2
Sigma70-like	0	0	4	4
SOH1	0	0	2	2
SRS	0	0	2	2
TAZ	3	0	11	10
TUB	0	0	15	14
zf-HD	0	0	10	11
CSD	1	1	5	5
GeBP	0	1	5	6
LOB	1	1	9	9
RB	0	1	2	2
Rcd1-like	0	1	3	4
RWP-RK	1	1	15	15
SBP	0	1	7	7
SNF2	1	1	15	14
SWI/SNF-SWI3	0	1	2	2
TCP	1	1	11	10
Trihelix	0	1	31	29
VOZ	0	1	5	5
ARF	0	2	10	9
BES1	1	2	13	12
Jumonji	0	2	10	10
TRAF	0	2	10	10
E2F-DP	2	3	6	6
GRF	1	3	6	6
HMG	1	3	11	11
MADS	0	3	16	14
C3H	0	4	57	57
Pseudo ARR-B	0	4	8	8
SET	2	4	10	10
C2C2-CO-like	0	5	14	13
AUX/IAA	1	6	25	26
GRAS	2	6	34	36
bHLH	4	7	59	59
C2C2-Dof	1	7	24	24
CCAAT	1	7	39	40
G2-like	1	7	37	37
HSF	0	7	38	37
PHD	1	7	16	15
SWI/SNF-BAF60b	1	7	6	6
Orphans	2	8	48	48
bZIP	3	10	74	75
WRKY	3	11	70	68
Tify	1	12	28	25
C2H2	2	15	47	49
NAC	4	16	81	80
HB	2	19	51	49
MYB	11	19	119	116
AP2-EREBP	10	37	131	124

## Data Availability

The data presented in this study are available in this article and its Supplementary Materials.

## Supplementary Materials

The following supporting information can be downloaded at: https://www.mdpi.com/article/10.3390/ijms241310641/s1.
