# Supplementary material for: Transcriptomic Profiling Highlights the ABA Response Role of BnSIP1-1 in Brassica napus
Source: Int J Mol Sci. 2023 Jun 26;24(13):10641. doi: 10.3390/ijms241310641 (PMC10342154; doi:10.3390/ijms241310641)
Supplement: Supplementary file 1 [file ijms-24-10641-s001.zip › Figure S1.pdf]

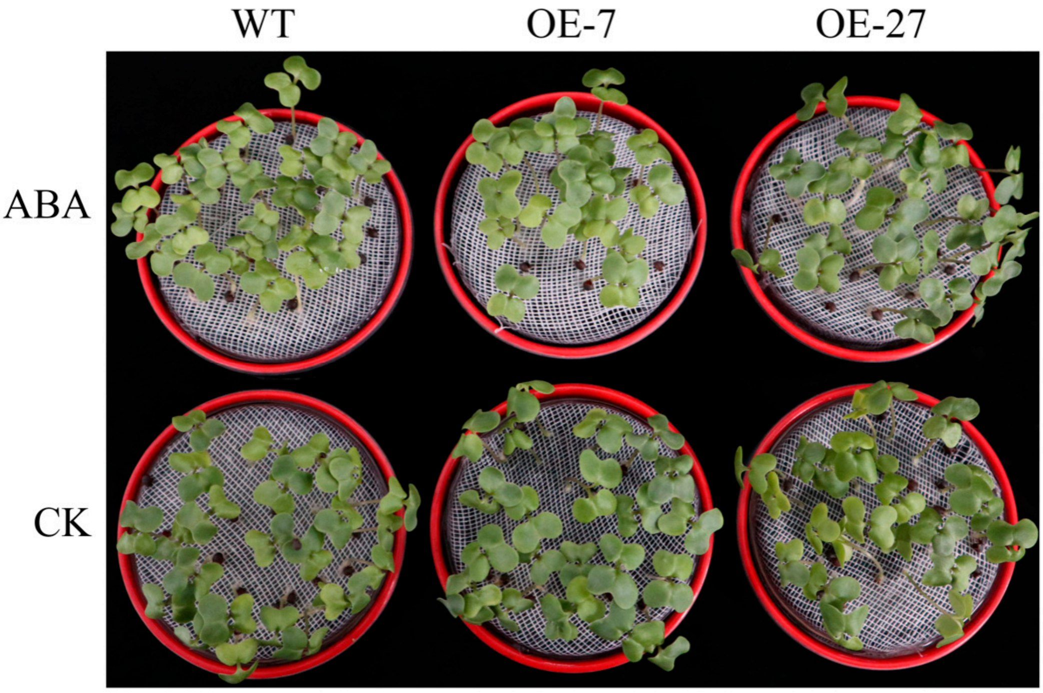

Figure S1. Growth of four-days-old wild type and *BnSIP1-1* transgenic seedlings with or without 10 mM ABA treatment. This photo was taken after ABA treatment for 6h.
