# Supplementary material for: Transcriptomic Profiling Highlights the ABA Response Role of BnSIP1-1 in Brassica napus
Source: Int J Mol Sci. 2023 Jun 26;24(13):10641. doi: 10.3390/ijms241310641 (PMC10342154; doi:10.3390/ijms241310641)
Supplement: Supplementary file 1 [file ijms-24-10641-s001.zip › Figure S2.pdf]

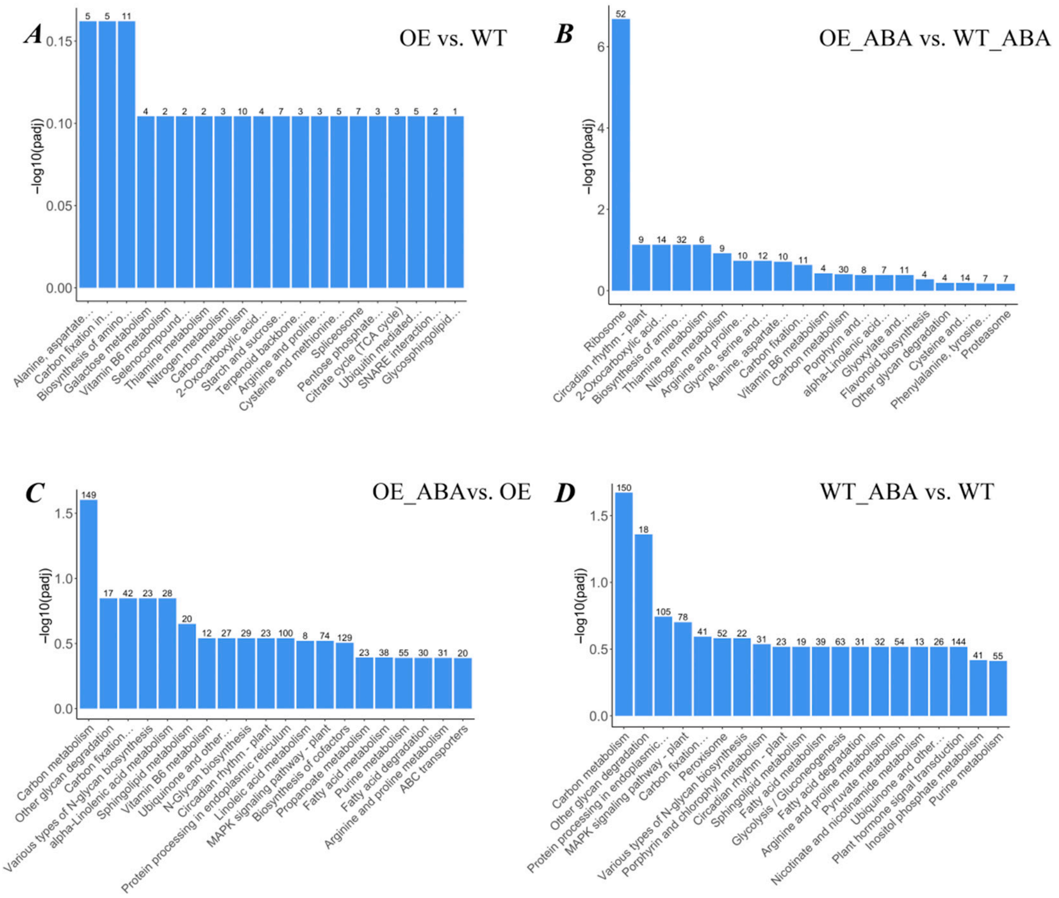

Figure S2. KEGG enrichment bar plot of DEGs among 4 comparison groups. The vertical axis represents the negative logarithm of P-value. The horizontal axis represents the name of pathway, the number among each bar represents the number of DEGs annotated to this pathway.(A)-(D) the bar plot of most enriched KEGG terms among 4 comparison groups.
